# Injectable Chitosan Hydrogel Particles as Nasal Packing Materials After Endoscopic Sinus Surgery for Treatment of Chronic Sinusitis

**DOI:** 10.3390/gels11010060

**Published:** 2025-01-11

**Authors:** Yusuke Yamashita, Kei Hosoya, Yukio Fujiwara, Yoichi Saito, Masahiro Yoshida, Shoji Matsune, Kimihiro Okubo, Takayuki Takei

**Affiliations:** 1 Department of Chemical Engineering, Graduate School of Science and Engineering, Kagoshima University, 1-21-40 Korimoto, Kagoshima 890-0065, Japan; k5290028@kadai.jp (Y.Y.); myoshida@cen.kagoshima-u.ac.jp (M.Y.); 2 Nose and Smell Clinic Ikebukuro, Tokyo 171-0022, Japan; 3 Department of Otolaryngology, Musashi Kosugi Hospital, Nippon Medical School, Kanagawa 211-8533, Japan; sm1988@nms.ac.jp; 4 Department of Cell Pathology, Graduate School of Medical Sciences, Faculty of Life Sciences, Kumamoto University, 1-1-1 Honjo, Chuo-ku, Kumamoto 860-8556, Japan; fuji-y@kumamoto-u.ac.jp; 5 Laboratory of Bioengineering, Faculty of Advanced Science and Technology, Kumamoto University, 2-39-1 Kurokami, Chuo-ku, Kumamoto 860-8555, Japan; west-east@mech.kumamoto-u.ac.jp; 6 Department of Otorhinolaryngology, Nippon Medical School, Tokyo 113-8602, Japan; ent-kimi@nms.ac.jp

**Keywords:** endoscopic sinus surgery, chronic sinusitis, nasal packing, chitosan, hydrogel, hemostasis, wound healing

## Abstract

After endoscopic sinus surgery (ESS), nasal packing is often used to stop bleeding and promote wound healing. Because maintaining a moist environment is important to enhance wound healing, hydrogel-based wound dressings are effective to promote wound healing. Chitosan is used in the medical field because of its high hemostatic and wound healing properties. We developed a pH-neutral and non-toxic chitosan hydrogel, which was difficult to achieve using conventional methods. In this study, we show in animal experiments that the chitosan hydrogel (hydrogel particles) had higher wound healing properties than a commercially available solid wound dressing (dry state) composed of the same polymer. Additionally, we applied the injectable chitosan hydrogel particles as nasal packing materials to patients with bilateral chronic sinusitis undergoing ESS in a pilot clinical study. Concerning symptom scores, though the results narrowly missed statistical differences (*p* < 0.05), the average scores of our chitosan hydrogel were superior to those of a commercially available wound dressing (especially *p* = 0.09 for nasal bleeding). These findings suggest that the injectable chitosan hydrogel could be a viable option as a packing material following ESS.

## 1. Introduction

Endoscopic sinus surgery (ESS) is the gold standard treatment for chronic sinusitis that fails to improve with drug therapy, and it is less burdensome to patients [1]. For several days after ESS, patients suffer from bleeding from the nasal mucosa and resected site. Nasal packing materials are often used for hemostasis and wound healing after ESS [2,3]. Generally, it is important to maintain a moist environment in the wound to enhance wound healing [4]. In a moist environment, epithelial cells migrate rapidly and collagen production is enhanced [5]. Hydrogels have often been used for wound care because they moisten wounds well. These hydrogels are made from non-bioabsorbable or bioabsorbable polymers [6,7].

Chitosan is a polysaccharide obtained by the alkali-based deacetylation of chitin, a natural polysaccharide that constitutes the exoskeleton of crustaceans [8]. The polymer is used in the medical field for its high biocompatibility, bioabsorbability, antimicrobial activities, hemostatic properties [9], and wound healing properties [10,11]. In the past, the medical application of chitosan hydrogels has not progressed because the polymers are only soluble in acidic solvents, making chitosan hydrogels acidic [12]. In addition, additives such as chemical cross-linkers [13,14] and polymerization initiators [15], which are highly biotoxic, were required for preparing chitosan-based hydrogels. Therefore, these chitosan hydrogels have not been used for medical applications.

On the other hand, our previous research found that chitosan–gluconic acid conjugate (CG) can dissolve in pH-neutral water and be gelled simply by freeze-thawing treatment, without adding any toxic additives [16]. In other words, the hydrogels are pH-neutral and contain no toxic chemicals. The biosafe chitosan hydrogels have good biological properties similar to unmodified chitosan, demonstrating excellent hemostatic and wound-healing properties. Furthermore, it has been shown that CG hydrogels are autoclavable, and autoclaving (steam sterilization) does not cause them to lose their excellent biological properties [17,18]. In the previous study, we demonstrated that there was no statistical difference between the wound-healing properties of the autoclaved CG hydrogel and those of the hydrogels disinfected with 70% ethanol. On the other hand, there has been no comparison of the wound-healing effect of dried-form chitosan/chitin versus chitosan hydrogel. It is important to compare the wound-healing properties of CG hydrogel with those of Beschitin-F^®^, a dry-state wound dressing composed of chitosan/chitin, to confirm its ability to promote moist healing. It is also important to evaluate the effectiveness of CG hydrogel as a nasal packing material in clinical studies. We have improved the CG hydrogel by crushing it into particles, making it injectable with a syringe. Injectable hydrogel is not only packed into the nasal cavity more easily than conventional sheet-based nasal packing materials, but it is also expected to provide seamless coverage of the wound surface.

In this study, the wound-healing properties of CG hydrogel were first compared with those of Beschitin-F^®^ through animal studies. Next, a pilot clinical study showed the potential of CG hydrogel as a nasal packing material.

Hydrogel particles are a form of colloidal particles. Colloidal particles such as liposomes and self-assembling coacervates have been reported as drug carriers [19,20]. CG hydrogel particles are bulk hydrogels (>1 mm in diameter). The CG hydrogels are degraded by lysozyme when they come into contact with body fluids. The degradation is expected to occur at the molecular level, ultimately breaking down into low-molecular-weight monomers. Therefore, its degradation products are expected to be smaller than those of previously reported liposomes [19] and self-assembling coacervates [20]. Regarding biological interactions, CG hydrogels have a positive charge in the body, causing their degradation products to adhere to negatively charged cell surfaces. In this respect, they are comparable to certain liposomes and self-assembling coacervates.

## 2. Results and Discussion

### 2.1. Preparation of CG Hydrogel Particle

CG hydrogels were prepared by freeze-thawing of pH-neutral CG aqueous solutions according to our previous report [16]. Then, the hydrogels were pulverized by high-speed agitation to form particles. Finally, the hydrogel particles were autoclaved (121 °C, 20 min) for sterilization. Since the hydrogel particles are produced only by physical crushing of our developed hydrogel (autoclaved CG hydrogel) [17], their crystal and chemical structures are expected to be equal to those of uncrushed hydrogel.

**Figure 1:**
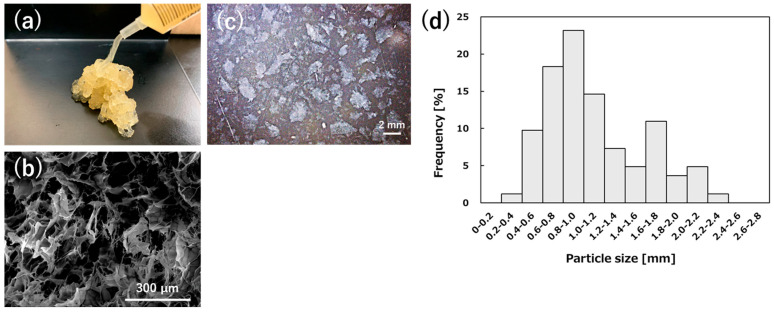
(**a**) The appearance of the CG hydrogel particles, (**b**) observation of the hydrogel with scanning electron microscope (scale bar: 300 μm), (**c**) observation of the hydrogel with stereo microscope (scale bar: 2 mm), and (**d**) particle size distribution of CG hydrogel particles (n = 82).

Figure 1a shows the appearance of the CG hydrogel particles after autoclaving. The hydrogel particle sample in the syringe could easily be pushed out with one hand. The ejected hydrogel particles retained their extruded shape. This property is expected to prevent it from falling out easily when filled into the nasal cavity. The micromorphology of CG hydrogel particles was observed by scanning electron microscope (SEM). As shown in Figure 1b, the porous structure is clearly formed of interconnected pores. The interpenetrating porous structure is expected to facilitate the transport of oxygen, nutrients, and growth factors necessary for wound healing. CG hydrogel particles were observed by an optical microscope and were irregular in shape. The particle size distribution is shown in Figure 1d. The hydrogel particles had an average size of 1.1 ± 0.5 mm, ranging from 0.4 to 2.2 mm. The size is considered to allow for easy extrusion by syringe. However, the CG hydrogel particles need to be optimized in terms of stability and degradability in the body in future studies.

### 2.2. Comparison of CG Hydrogels with Beschitin-F by Animal Experiments

**Figure 2:**
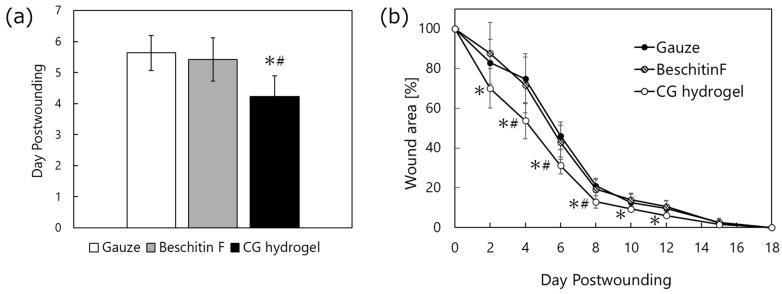
The results of the treatment of wounds in the dorsal skin of rats with gauze, Beschitin-F^®^, and CG hydrogel. (**a**) The number of days required for the wound area to shrink to 50% of the area at the time of injury. (**b**) The reduction in the wound area (n = 4–8). * *p* < 0.05 vs. gauze and Beschitin-F^®^, # *p* < 0.05 vs. Beschitin-F^®^.

**Figure 3:**
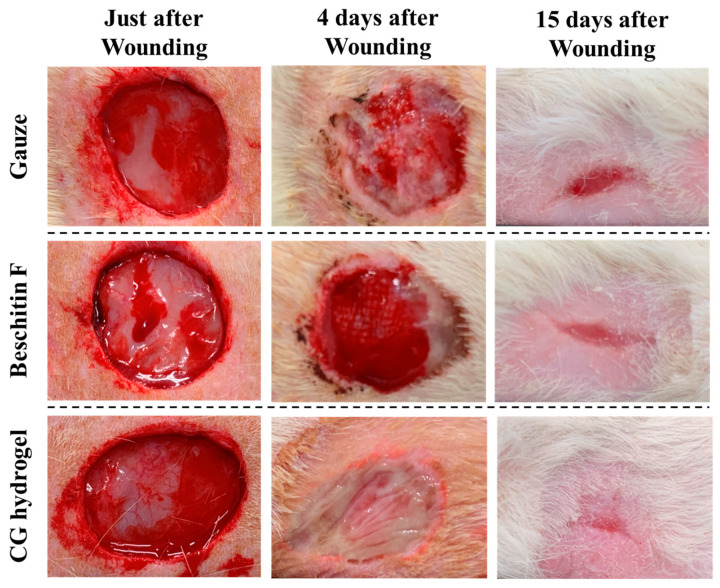
Regeneration and healing process of wounds created on the dorsal skin of rats and treated with gauze, Beschitin-F^®^, and CG hydrogels at 0 days (just after wounding), 4 days, and 15 days after wounding.

In the animal experiment, we compared the wound-healing properties of CG hydrogel (moist healing) and Beschitin-F^®^ (dry healing, chitin/chitosan-coated gauze). We used a rat model of full-thickness skin defects to validate more clearly the differences in wound healing properties between these chitosan materials. Figure 2a shows the time required for the wound area covered with gauze, Beschitin-F^®^, and CG hydrogel to shrink to 50% of the initial area. The wounds treated with gauze, Beschitin F^®^, and CG hydrogel healed to 50% of their initial area over 5.6 ± 0.6, 5.4 ± 0.7, and 4.2 ± 0.7 days, respectively. Figure 2b shows the reduction in the area of wounds covered with each material. The wound regeneration process is also shown in Figure 3. CG hydrogel significantly accelerated wound healing compared to other wound dressings (analysis of variance (ANOVA), Bonferroni method). These results indicate that CG hydrogel is the most effective in promoting wound healing among the tested materials. The reason CG hydrogel was able to heal wounds faster than Beschitin-F^®^ is attributed to the synergistic effects of moist healing by the large amount of water contained in CG hydrogel and the wound-healing effect of the polymer itself.

**Figure 4:**
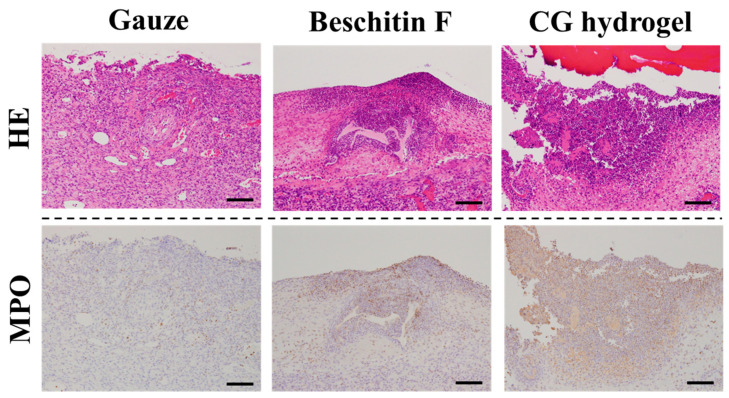
Hematoxylin and eosin (HE)- and immunohistochemically stained sections of myeloperoxidase (MPO) of wound tissues treated with gauze, Beschitin-F^®^, and CG hydrogels on day 6 after wounding. Scale bars are 100 µm.

Figure 4 shows the histological staining of wound cross-sections treated with gauze, Beschitin-F^®^, and CG hydrogel. MPO-positive neutrophil infiltration was stronger with Beschitin-F^®^ and CG hydrogel compared to gauze and tended to be stronger with CG hydrogel than with Beschitin-F^®^. Chitosan is reported to be degraded by hydrolytic enzymes in the body, releasing degradation products that attract neutrophils [10]. The CG hydrogels were degraded by lysozyme, which is an enzyme in the body, as well as chitosan in our previous studies [16,18]. We hypothesize that the CG hydrogel exhibited stronger neutrophil infiltration than the solid Beschitin-F^®^ because it was more easily degraded, releasing more degradation products. It has been reported that infiltrating neutrophils secrete liquid factors that promote wound healing [10], explaining why chitin and chitosan-based wound dressings are effective.

### 2.3. Treatment of Wounds in Sinuses After ESS Using CG Hydrogel in Pilot Clinical Studies

Here, we used Kaltostat^®^ as a comparison for our CG hydrogels. Kaltostat^®^ is a soft, nonwoven dressing composed of calcium sodium alginate fibers [21]. Upon contact with a wound, it absorbs exudate, initiating an ion exchange between the calcium in the dressing and the sodium in the wound exudate. This process gradually converts the dry dressing into a firm, moist hydrogel. Kaltostat^®^ is widely used for the hemostasis of open wounds and as a packing material for the paranasal sinuses following ESS [22].

Table 1 illustrates the baseline characteristics of patients in the CG hydrogel and Kaltostat^®^ groups. Among the CG hydrogel group (n = 3), the gender distribution was 1 female to 2 males, whereas the Kaltostat^®^ group (n = 4) had an equal distribution of 2 females to 2 males (*p* = 0.72). There was no significant difference in severity and type of sinusitis between the two groups.

**Figure 5:**
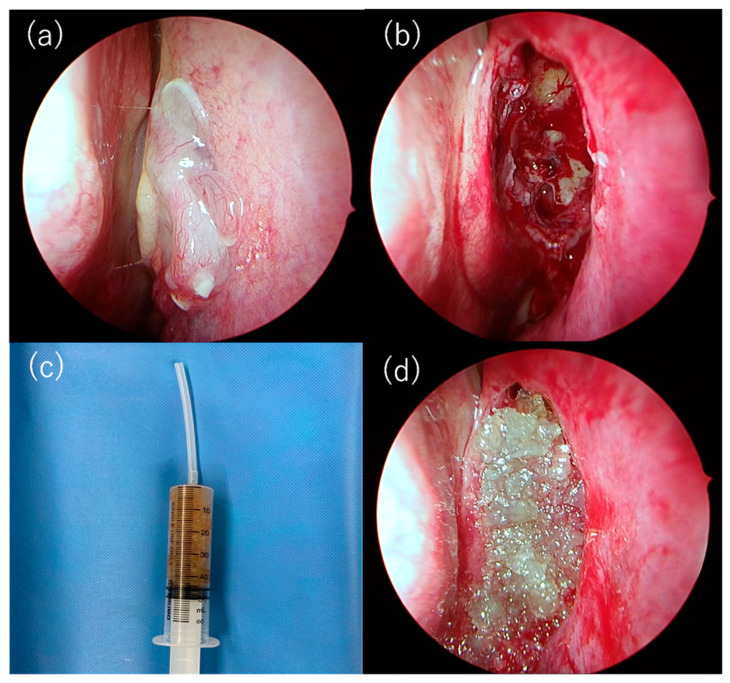
(**a**) Preoperative findings in the left nasal cavity; (**b**) post-ESS; (**c**) CG hydrogel filled in the syringe; and (**d**) sinuses after filling with hydrogel.

Figure 5 shows the appearance of the nasal cavity before and after the procedure, as well as CG hydrogels. Using syringes, the CG hydrogels were successfully administered into the wound created by the surgery.

**Figure 6:**
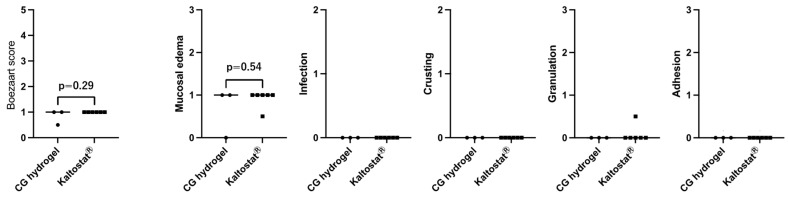
Boezaart scores and wound healing scores. The bars are presented as the median and interquartile range (IQR).

**Figure 7:**
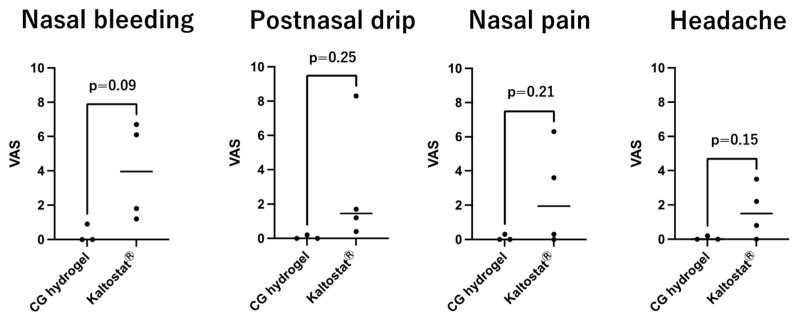
Symptom scores (VAS 0–10) reported by patients on the day after surgery. The bars are presented as the median and interquartile range (IQR).

Figure 6 and Figure 7 show Boezaart and wound healing, as well as symptom scores (visual analog scores (VAS) reported by patients). The lower scores indicate more favorable results in these figures. There was no significant difference between CG hydrogel and Kaltostat^®^ in Boezaart and wound healing scores. Concerning symptom scores, though the results narrowly missed statistical differences (*p* < 0.05), the average scores of CG hydrogel shown in Figure 7 were superior to those of Kaltostat^®^. In particular, despite the quite small sample number (n = 3 and 4), a *p*-value of 0.09 for the nasal bleeding score was achieved. Reduction in nasal bleeding is one of the most important properties of nasal packing materials. Considering that Kaltostat^®^ is known to be one of the best nasal packing materials in terms of hemostasis, the superiority of CG hydrogel to Kaltostat in nasal bleeding is an important result. Some of the amino groups of chitosan are protonated in water, which causes the polymer to become positively charged. The positively charged chitosan or CG binds electrostatically to negatively charged red blood cells and platelets, promoting aggregation. In our previous study, CG hydrogel also showed high hemostatic effect in vitro in blood clotting tests [17]. It should also be noted that the patient’s nose was less painful. Chitosan has been reported to reduce inflammation and pain [23,24]. The lower scores for nasal pain in this study could be attributed to the pain suppression effect of chitosan. These findings suggest that CG hydrogel could be a viable option as a packing material following ESS.

CG hydrogels are considered to have a significant advantage in that they are injectable. Conventional nasal packing materials are made of gauze or sponge, and do not adequately cover the wound surface. In addition, the removal of these packing materials sometimes causes pain and secondary wounds [25,26]. In contrast, as shown in Figure 5c, CG hydrogel can be packed seamlessly into the nasal cavity with a syringe and a tube. Moreover, the hydrogel can be removed by nasal rinse, resulting in less pain and a lower risk of secondary wounds. Furthermore, it is expected that hydrogels filling the nasal cavity also block bacterial invasion. The injectability of the CG hydrogel demonstrates its excellent properties and utility as a nasal packing material.

The usefulness of other chitosan hydrogels as a packing material has been previously reported [27,28]. One report lacked detail on the preparation method of chitosan hydrogel, and may not have addressed the issues of conventional chitosan hydrogels being acidic and containing toxic chemicals. On the other hand, the CG hydrogels in this study can solve those problems. In the second paper, we interpret that the authors considered a high-concentration aqueous carboxymethylated chitosan solution as a hydrogel. The highly concentrated aqueous chitosan solution is neutral and contains no toxic chemicals, and therefore we guess that it solves the problem of conventional chitosan hydrogel. However, since the solution is injected into the nasal cavity with a syringe, it is considered that it has not lost its fluidity. Thus, it may flow down from the affected area. On the other hand, our hydrogels are completely gelled and are expected to reduce such leakage from the affected area.

## 3. Conclusions

In this study, the efficacy of injectable CG hydrogel particles was assessed through both an animal experiment and human clinical studies. We examined the wound healing of full-thickness skin defects created on rat skin. Wound healing was significantly enhanced with the CG hydrogel particles in comparison with gauze and a commercially available solid chitosan/chitin-based wound dressing. In a clinical study, concerning symptom scores, though the results narrowly missed statistical differences (*p* < 0.05), the average scores of our CG hydrogel were superior to those of a commercially available wound dressing (in particular, *p* = 0.09 for nasal bleeding despite the small sample number (n = 3 and 4)). These findings suggest that the injectable chitosan hydrogel could be a viable option as a packing material following ESS. The injectable CG hydrogel particles developed in this study, initially designed for wound healing after ESS, have significant potential for broader applications in treating wounds of other parts of the body. For example, they can be applied in the regeneration of missing periodontal tissue after periodontal surgery or to the treatment of bedsores, offering their versatility in various medical applications. In this study, CG hydrogel particles were used for wound healing after ESS; however, some properties, such as the gluconic acid content of CG and particle size, were not optimized. In future studies, we will focus on optimizing CG hydrogel particles and evaluating their effects on wound healing properties.

## 4. Materials and Methods

### 4.1. Materials

Chitosan (deacetylation rate: 83%, viscosity average molecular weight: 2.3 × 10^5^ Da) was purchased from KIMICA Corporation (Tokyo, Japan). Sodium gluconate and N-hydroxysuccinimide (NHS) were purchased from Wako Pure Chemical Corporation (Tokyo, Japan). 1-(3-Dimethylaminopropyl)-3-ethylcarbodiimide (EDC) was purchased from Peptide Institute, Inc. (Osaka, Japan). 2-(N-morpholino)ethanesulfonic acid (MES) was purchased from Dojindo Laboratories (Kumamoto, Japan).

### 4.2. Synthesis of CG

CG was synthesized by condensing the carboxyl group of gluconic acid to the amino group of chitosan using carbodiimide chemistry utilizing EDC and NHS, as described in our previous report [16]. Chitosan (5.0 g) was dissolved in 500 mL of 25 mM MES aqueous solution, and the pH was adjusted to 4 with HCl. Sodium gluconate (1.35 g), EDC (1.18 g), and NHS (0.37 g) were then dissolved in the MES aqueous solution. The mixture was stirred at 30 °C for 24 h. After that, the pH was adjusted to 8.0 with 6 M NaOH, followed by the addition of 2500 mL of 99% (*v*/*v*) ethanol to precipitate the product. The precipitates were collected by vacuum filtration and dialyzed in deionized water using a dialysis membrane with a molecular weight of 14,000 fractions (Wako Pure Chemical, Tokyo, Japan). Dialysis was repeated until the electrical conductivity of the dialysate matched that of the deionized water. The purified CG was lyophilized to obtain a spongy form, which was subsequently ground into powder. The gluconic acid content (the number of gluconic acids modified per 100 glucosamine units of chitosan) was determined by colloidal titration.

### 4.3. Preparation of CG Hydrogel

CG (gluconic acid content: 8%) was dissolved in dilute hydrochloric acid to a concentration of 2% (*w*/*v*) (pH 4.0). The pH was adjusted to 7.0 by adding 0.1–1.0 M NaOH aqueous solution dropwise, while the solution was stirred with a magnetic stirrer. Hydrogels were prepared by freezing the neutralized CG solution at −20 °C for 6 h, followed by thawing at room temperature for 2 h. The hydrogel was then placed in a glass flask containing excess saline solution and crushed by stirring at 1500 rpm for 30 min using a double-blade mixer. The resulting suspension was centrifuged at 3500 rpm for 5 min to collect the hydrogel particles.

### 4.4. Animal Expriments

Under anesthesia with isoflurane, male Wistar rats (10 weeks old, Nippon Clare) were injected intraperitoneally with a saline solution containing streptozotocin (10 mg/mL) at 50 mg-streptozotocin/kg-rats, followed by a second injection two weeks later. Two weeks after the second streptozotocin injection, blood glucose levels were measured, and rats with blood glucose levels above 204 mg/dL were used in the experiment. The rats were intraperitoneally injected with a triad of anesthetics (medetomidine hydrochloride, midazolam, and butorphanol tartrate) [29]. The rats’ backs were disinfected with 70% ethanol and then shaved. Using surgical scissors, three 2 cm diameter wounds (circular skin defects) were created on the dorsal skin of rats. Each of the three wounds was covered with gauze, Beschitin-F^®^ (Nichiban, Japan), and CG hydrogel. A waterproof film (BFR, Nichiban, Japan) was applied over them. The rats’ torsos were wrapped with an adhesive bandage (Skinergate Gachitt SGG75, Nichiban, Japan) to secure the hydrogels. Every two days, under anesthesia with isoflurane, the dressings were replaced with new ones. At the time of the exchange, the wounds were traced on a transparent sheet and the wound areas were determined using image processing software (ImageJ (version 1.54g)). For tissue staining of wound cross-sections, rats were euthanized under isoflurane overdose, and the wounds and their surrounding tissue were harvested and soaked in a 10% neutral buffered formalin solution.

### 4.5. Histological Analysis

After formalin (10% neutral buffered) fixation, rat wound tissue was paraffin-embedded, thinly sliced at 3 µm thickness, and stained with hematoxylin and eosin (HE). For the immunostaining of myeloperoxidase (MPO), thin sections were microwaved with 1 mM EDTA (pH 8) solution for antigen activation, then treated with the primary antibody (anti-MPO antibody (A0398, Dako (Santa Clara, CA, USA)) and secondary antibody (anti-rabbit antibody (MAX-PO(R)) for rat tissue, Nichirei Bioscience Inc., Tokyo, Japan. The 3,3′-diaminobenzidine (DAB) substrate kits (Nichirei Bioscience Inc., Tokyo, Japan) were used for color development.

### 4.6. Pilot Clinical Studies

The sinuses and common nasal passages of three patients, who gave consent for ESS for chronic sinusitis, were packed with CG hydrogels. Surgery was performed on patients over 20 years old with no history of crustacean allergy. The control group consisted of patients who underwent ESS under general anesthesia at our hospital between January and March 2021, where other packing materials were used. The following characteristics of the cases were recorded from the medical records: gender, age, nasal polyp score, preoperative Lund–Mackay CT scores, Eosinophilic Chronic Rhinosinusitis (ECRS) [30], peripheral eosinophil count, total IgE, asthma, and allergic rhinitis. The study was approved by the ethics committee of Nippon Medical School Tama Nagayama Hospital (approval number 680). CG hydrogels were inserted into the middle and common nasal passages using a syringe after ESS (Figure 5). Kaltostat^®^ (7.5 × 12 cm) was divided into 4 sections and placed within a total of 2 sheets in the middle and common nasal passages [31]. The packing in the common nasal passage was removed on the second postoperative day. Cefazolin sodium was administered intravenously at a dose of 2 g/day on the day of surgery, followed by oral administration of cefditoren pivoxil at 300 mg/day for five days postoperatively. Acetaminophen injection 1000 mg was used for pain on the day of surgery if requested by the patient. Nasal gargling started 3–6 days postoperatively.

The day after surgery, the patients were given a document to record their visual analog scale (VAS) score (0–10) for nasal pain, headache, bleeding, and postnasal drip. Packing materials were removed and the nasal lining was cleaned. Sinus conditions were checked endoscopically, and bleeding was assessed endoscopically using the six-point Boezaart score [32], where 0 indicated no bleeding and 5 indicated severe bleeding after the packing material was removed. Wound healing score was recorded separately for mucosal edema (0–3), infection (0–2), crushing (0–2), granulations (0–3), and adhesion (0–3) [28]. The Boezaart and wound healing scores were assessed by two surgeons using the photographs taken, and the average of the left and right sides was calculated.

### 4.7. Statistical Analysis

The results of the animal experiment were statistically analyzed using analysis of variance (ANOVA, Bonferroni method), with *p* < 0.05 considered statistical significance. The data from the human clinical study were presented as the median and interquartile range (IQR). Comparisons between the two groups were performed using the Mann–Whitney test and Fisher’s exact test, with *p* < 0.05 (two-sided) considered statistical significance.

## Figures and Tables

**Table 1:** Clinical characteristics of the study patients.

	CG Hydrogel (n = 3)	Kaltostat^®^ (n = 4)	*p*-Value
Gender, female–male, n	1:2	2:2	0.72
Age, years (median, (IQR))	55.0 (55.0–63.0)	44.0 (36.5–54.3)	0.20
Total polyp score (median, (IQR))	4.0 (3.0–5.0)	1.0 (0–3.0)	0.35
CT scores (median, (IQR))	15.0 (14.0–17.0)	12.0 (10.5–13.8)	0.20
ECRS (n, %)	3 (100)	3 (75.0)	0.44
Peripheral eosinophil count, % (median, (IQR))	9.9 (9.5–10.1)	4.8 (3.7–5.7)	0.02
Total IgE, IU/mL (median, (IQR))	186 (181.5–321.0)	38.0 (34.3–57.8)	0.04
Septorhinoplasty (n, %)	3	4	-
Submucosal inferior turbinectomy (n, %)	3	4	-
Asthma (n, %)	2 (66.7)	0 (0)	0.06
Allergic rhinitis (n, %)	3 (100)	2 (50.0)	0.20

Data are expressed as medians (IQR). IQR, interquartile range; CT, computed tomography; ECRS; eosinophilic chronic rhinosinusitis; statistical significance at *p* < 0.05 with the Mann–Whitney U or Fisher’s exact test.

## Data Availability

Data presented in this study are available on request from the corresponding authors.
